# Public health round-up

**DOI:** 10.2471/BLT.20.010920

**Published:** 2020-09-01

**Beirut explosion**

Just after 6pm on Tuesday, 4 August, a massive explosion of ammonium nitrate stored at the main port in Beirut, Lebanon, killed at least 150 people and injured more than 6000. Hospitals and clinics were also damaged, compromising a health system that was already under strain. The World Health Organization sent 20 tonnes of health supplies to the city on 5 August as part of a broader response.

**Figure:**
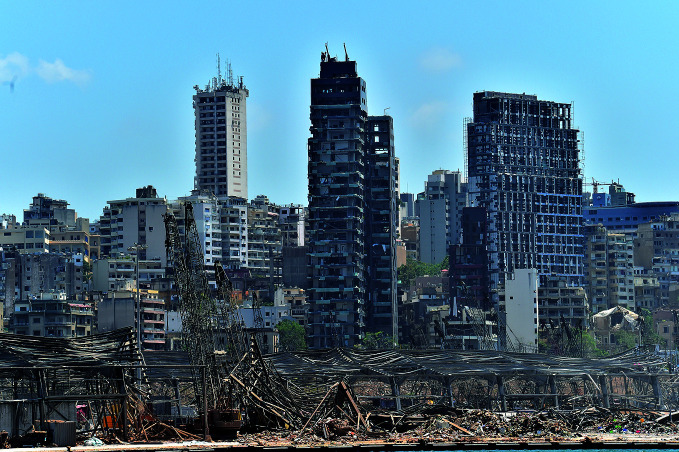
UNICEF/Ocha

## Ebola spreading

The Ebola virus disease outbreak in the Democratic Republic of the Congo is spreading, with cases being confirmed in new locations in Équateur Province, the focus of the outbreak. As of 8 August, eight of the 18 health zones in the province were reporting infections, six of which between 1-8 August.

Since the start of the current outbreak, which was declared in June based on genetic analysis of the virus in circulation, a total of 79 people had been infected by 8 August, 33 of whom had died.

A number of challenges are hampering response efforts, including community resistance to blood sampling and recommended safe burial practices. The continuing presence of infected people living in the community and contacts being lost to follow-up are of serious concern.

The response is also being complicated by an increase in COVID-19 cases, a long-standing measles outbreak and a complex ongoing humanitarian crisis.

Urgent action is required to limit further spread, including intense community engagement to ensure compliance with response measures. Partners also need urgently to address the lack of funding for the response.

https://bit.ly/3fQcHsj

## Beirut explosion

The massive explosion of stored ammonium nitrate that occurred in Beirut’s main port on 4 August killed over 150 people and injured more than 6000, including over 130 who were admitted to intensive care units. An estimated 300 000 people were left homeless.

Five hospitals in the area were also badly damaged as were many health centres and primary health care facilities in the city. As a result, patients were transferred to health facilities around the country.

The World Health Organization (WHO) sent 20 tonnes of health supplies to Beirut on 5 August. The supplies were airlifted from WHO’s logistics hub in Dubai, using a plane donated by the Government of the United Arab Emirates, and were sent to hospitals receiving and treating injured patients.

The supplies were enough to cover 1000 trauma interventions and 1000 surgical interventions. Further supplies and equipment were urgently required, including for hospital emergency departments and intensive care units.

WHO officials also worked with national health authorities to assess the public health impact of the blast and focused on ensuring the continuity of the country’s COVID-19 response. This includes resupplying stocks of personal protective equipment that was destroyed in the explosion.

https://bit.ly/2E0NNZu

## A protracted pandemic

The 2019 novel coronavirus disease (COVID-19) pandemic will require sustained, long-term response efforts at the community, national, regional, and global levels. This was the main conclusion of the International Health Regulations Emergency Committee on COVID-19, which held its fourth meeting on 31 July.

Tedros Adhanom Ghebreyesus, WHO Director-General, shared the committee’s view, stating, “Many countries that believed they were past the worst are now grappling with new outbreaks. Some that were less affected in the earliest weeks are now seeing escalating numbers of cases and deaths."

The committee advised the Director-General that the pandemic continues to constitute a public health emergency of international concern (PHEIC), an assessment he accepted.

The committee recommended that WHO continue to mobilize global and regional multilateral organizations and partners for COVID-19 preparedness and response, to support Member States in maintaining health services, while accelerating the research and eventual access to diagnostics, therapeutics, and vaccines.

It also called for countries to join initiatives, such as the Access to COVID-19 Tools Accelerator to ensure equitable allocation of approved COVID-19-relevant diagnostics, therapeutics and vaccines.

https://bit.ly/30S9aW7

## Hepatitis B progress

The proportion of children under five years chronically infected with the hepatitis B virus (HBV) dropped to just under 1% in 2019 down from around 5% in the pre-vaccine era, according to new estimates published by WHO.

The estimates were released on 27 July, World Hepatitis Day 2020. The decline in HBV infections marks the achievement of one of the targets to eliminate viral hepatitis set in the Sustainable Development Goals to reach under 1% prevalence of HBV infections in children under five years by 2020.

WHO called for united action to build on the achievement through intensified efforts to prevent mother-to-child transmission of HBV by testing pregnant women, providing antiviral prophylaxis to those who need it, and increasing access to hepatitis B immunization and birth dose vaccine.

https://bit.ly/30ROQnG

## School hand hygiene

Around two in every five schools lacked basic handwashing facilities at their schools in 2019.

This is according to data from a WHO and United Nations Childrens’ Fund (UNICEF) report released on 13 August.

Of the 818 million children lacking access to handwashing facilities, 355 million went to schools which had washing facilities with water, but no soap, and 462 million went to schools which had no facilities or water available for handwashing.

In the least developed countries, 7 out of 10 schools lack basic handwashing facilities and half of schools lack basic sanitation and water services.

The lack of handwashing facilities is a major concern for governments seeking to control the spread of COVID-19. However, as the report points out, it is vital that governments take into consideration the harm associated with prolonged school closures.

“We must prioritize children’s learning. This means making sure that schools are safe to reopen – including with access to hand hygiene, clean drinking water and safe sanitation.” said Henrietta Fore, UNICEF Executive Director, at the launch of the report.

https://bit.ly/2FkWIWx

**Cover Photo**

A young boy guides a herd of goats and sheep with his family in Rahim Yar Khan District in Punjab province, Pakistan.

**Figure:**
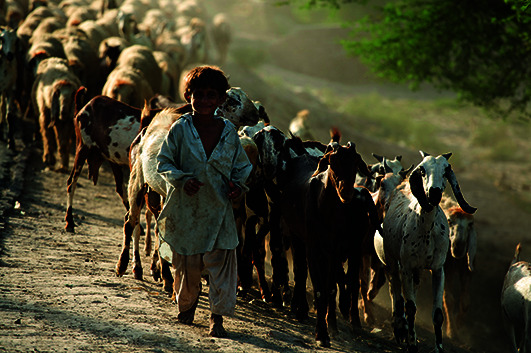
UNICEF/Shehzad Noorani

## Improving pandemic resilience

The WHO Regional Office for Europe (EURO) announced a Pan-European Commission on Health and Sustainable Development to rethink the region’s pandemic preparedness and response policies.

The commission is tasked with assessing the different ways in which different European countries have responded to the COVID-19 pandemic and will make recommendations on investments and reforms to improve the resilience of health and social care systems.

Announced 11 August, the commission was to start its work with an inaugural meeting on 26 August. EURO will act as secretariat to the commission and will support the production of a report which is to be presented by September 2021.

https://bit.ly/3kEp7qL

## Oral health guidance

WHO released interim guidance on the provision of oral health services during the COVID-19 pandemic.

Released on 3 August, the guidance is intended for public health authorities, chief dental officers at ministries of health and oral health care personnel working in private and public health sectors.

Aimed at reducing the risk of transmission, as much as possible, the guidance covers the prioritization of urgent cases, screening of patients, and measures to be taken in delivering oral health services.

Oral health provision involves physical proximity and uses equipment that generate aerosols. As a consequence, providers are at risk of being infected with COVID-19 and of passing the infection to patients.

https://bit.ly/2PLVoxF

## Virtual adoptions

The Member States of the World Health Organization (WHO) adopted a series of proposed World Health Assembly decisions generated through a ‘written silence procedure’ for the first time.

The decisions were proposed at the 73rd World Health Assembly, which took place on-line in May 2020 to reduce participants’ exposure to the novel coronavirus (SARS-CoV-2).

Under the written silence procedure, Member States receive a draft of a decision, specifying the deadline for raising an objection and allowing at least 72 hours for a response.

The decisions adopted relate to: strengthening global immunization efforts; cervical cancer prevention and control; a global strategy for tuberculosis research and innovation; prevention of vision impairment and blindness; strengthening efforts on food safety; a global strategy and plan of action on public health, innovation and intellectual property; a decade of healthy ageing; and influenza preparedness.

https://bit.ly/3h4MwiE

**Looking ahead**

September 10 - World Suicide Prevention day, 'Working Together to Prevent Suicide.' Online events. https://bit.ly/30rc1E7

September 14 – 15 - WHO Regional Committee for Europe; convened online. https://bit.ly/2Cd3XP1

September 17 - World Patient Safety Day, ‘Health Worker Safety: A Priority for Patient Safety.’ Online events. https://bit.ly/344qY2n

